# Accidental duodenal foreign body of toothbrush removed laparoscopically: a case report

**DOI:** 10.1186/s40792-022-01501-x

**Published:** 2022-07-27

**Authors:** Masahiro Soga, Tetsuya Tanaka, Takeshi Ueda, Yuki Kirihataya, Yohei Yamaguchi, Yasushi Okura, Masayoshi Sawai, Atsushi Yoshimura

**Affiliations:** 1 Department of Surgery, Minami-Nara General Medical Center, 8-1 Oaza-Fukugami Oyodo-cho Yoshino-gun, Nara, 638-8551 Japan; 2 Department of Gastroenterology, Minami-Nara General Medical Center, 8-1 Oaza-Fukugami Oyodo-cho Yoshino-gun, Nara, 638-8551 Japan

**Keywords:** Duodenum, Foreign body, Toothbrush, Laparoscopic surgery

## Abstract

**Background:**

Foreign body ingestion is a common case in daily medical care, and it usually passes through the entire gastrointestinal tract naturally and is excreted in the feces. However, long and sharp foreign bodies may be difficult to pass naturally due to their shape. Here, we present a rare case of a duodenal foreign body, a toothbrush, that required laparoscopic surgical removal after a failed endoscopic attempt.

**Case presentation:**

A 51-year-old male with intellectual disability presented to our hospital due to fever. Initially, he was diagnosed with aspiration pneumonia by chest X-ray and blood examination. However, abdominal X-ray examination suggested a foreign body, and a computed tomography scan revealed a toothbrush in the duodenum. Therefore, upper gastrointestinal endoscopy was immediately attempted to remove it, but it could not be safely removed because the handle part of the toothbrush seemed deeply embedded in the duodenal mucosa. Therefore, this case was diagnosed as duodenal incarceration of the toothbrush, and it was removed by laparoscopic surgery. The operation was performed safely, and the patient’s postoperative course was good without any complications. The extracted toothbrush was 15 cm in length.

**Conclusion:**

We experienced a rare case of a duodenal foreign body, which was a toothbrush. The duodenal foreign body was safely removed by laparoscopic surgery for the first time.

## Background

Foreign body ingestion is a common problem among all age groups. For children, most foreign body intakes are accidental; however, for adults, factors, such as mental illness, alcoholism, and bulimia nervosa, should be considered. The diagnosis is based on history and clinical and radiological examination; various foreign bodies can be seen on abdominal X-ray photographs in emergency departments. Most foreign bodies are relatively small, such as coins, buttons, and batteries. When foreign bodies are ingested, they naturally pass through the entire gastrointestinal tract and are excreted in the feces [1]. However, long and sharp foreign bodies may be difficult to pass naturally through the gastrointestinal tract due to their shape. Meanwhile, toothbrush is a rare foreign body to be ingested accidentally. Its unusual shape has no theoretical possibility of spontaneous passage. Therefore, unlike most other foreign bodies, there are no reports of swallowed toothbrush that passed naturally [2]. If foreign bodies do not pass through the gastrointestinal tract asymptomatically, well-known complications, such as pressure necrosis, perforation, bleeding, and gastrointestinal ulceration can occur, which can result in life-threatening sepsis. Therefore, an early removal is recommended to avoid these complications. Here, we present a rare case of a duodenal foreign body, which was a toothbrush, that required laparoscopic surgical removal after a failed endoscopic attempt.

## Case presentation

**Fig. 1:**
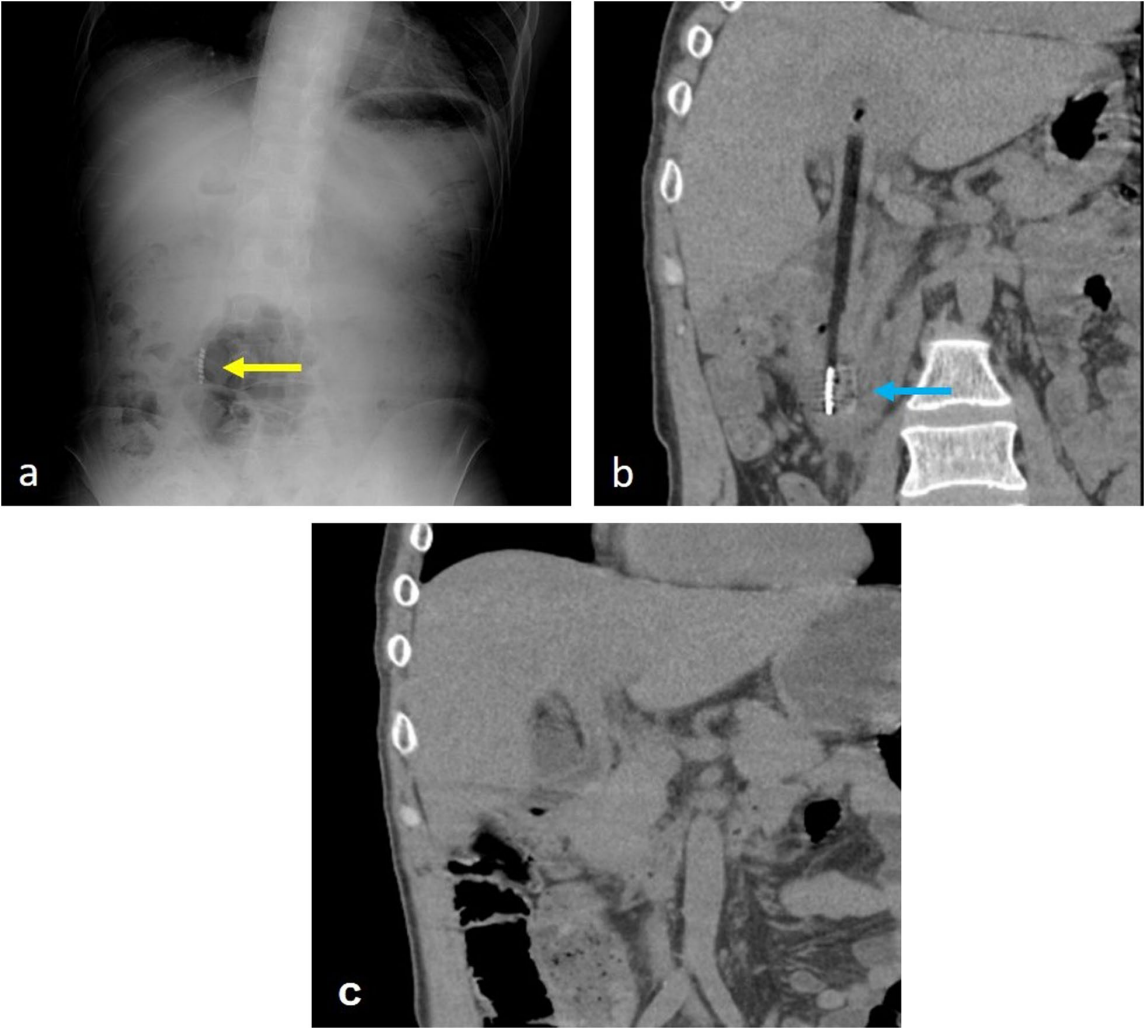
**a** A plain abdominal X-ray suggesting a foreign body (yellow arrow). **b** A computed tomography (CT) scan revealing a toothbrush in the duodenum (blue arrow). **c** A postoperative CT showing no changes in the liver

**Fig. 2:**
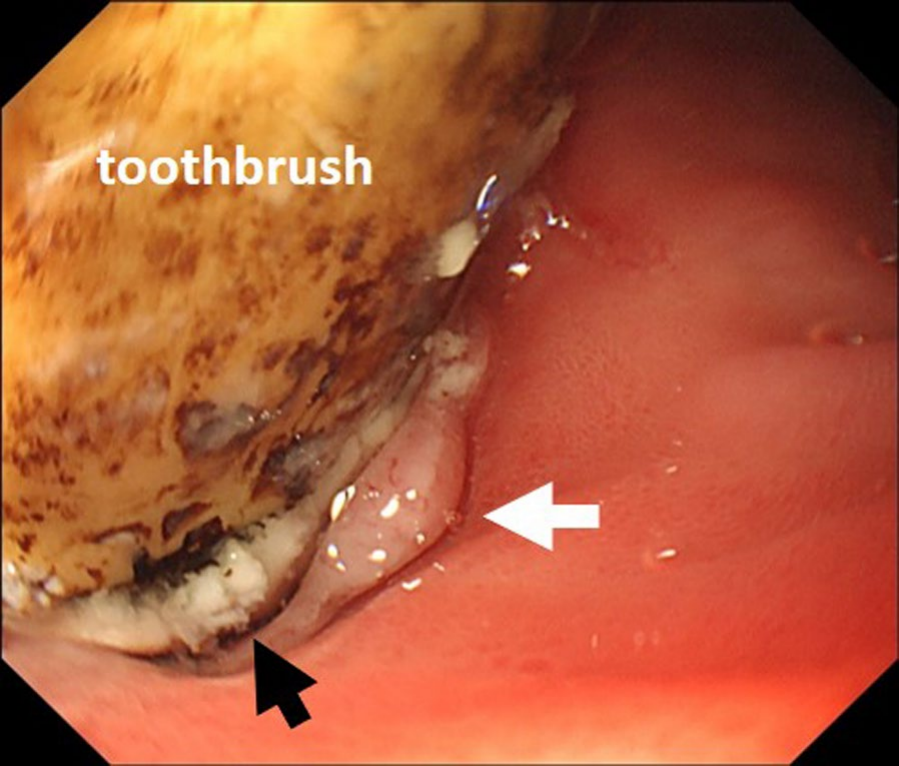
The toothbrush was embedded in the second part of the duodenum (black arrow), and there was a granuloma (white arrow) around the handle part of the toothbrush at the duodenal bulb

**Fig. 3:**
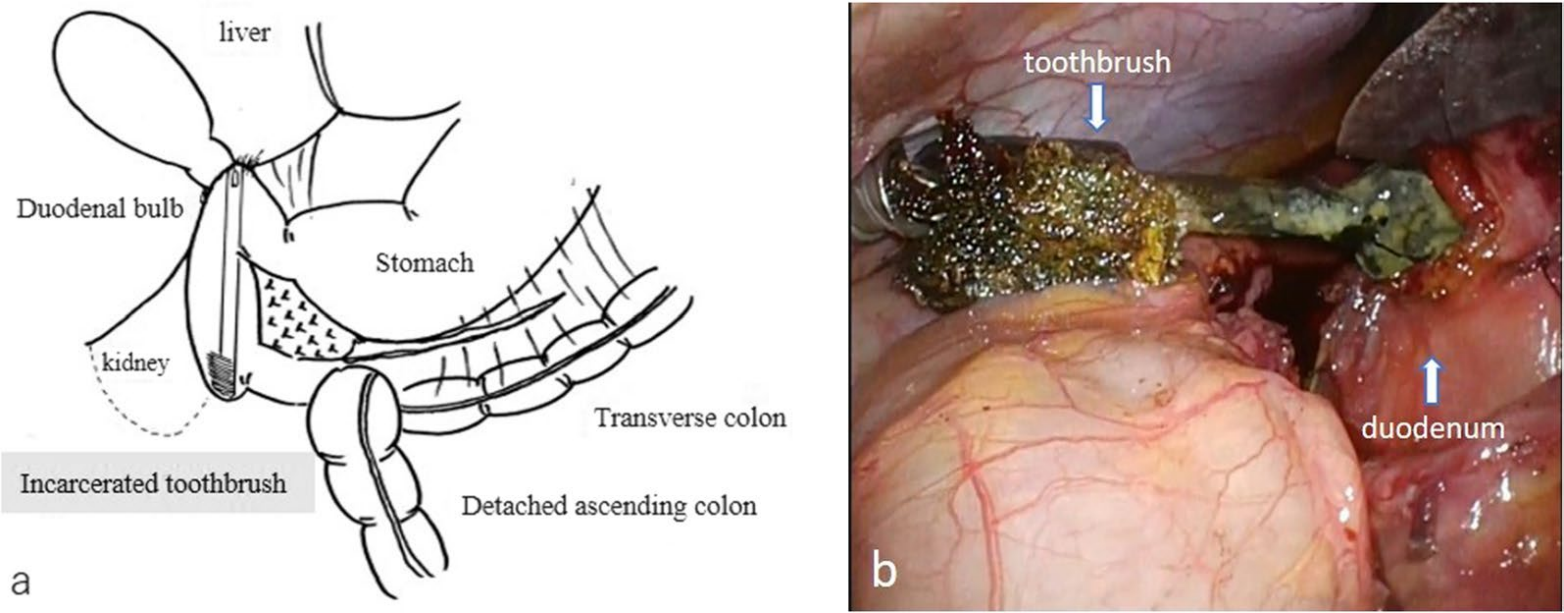
**a** Schema of surgical findings and approach. **b** Intraoperative photograph. The toothbrush was removed through the incision made on the caudal side of the duodenum

**Fig. 4:**
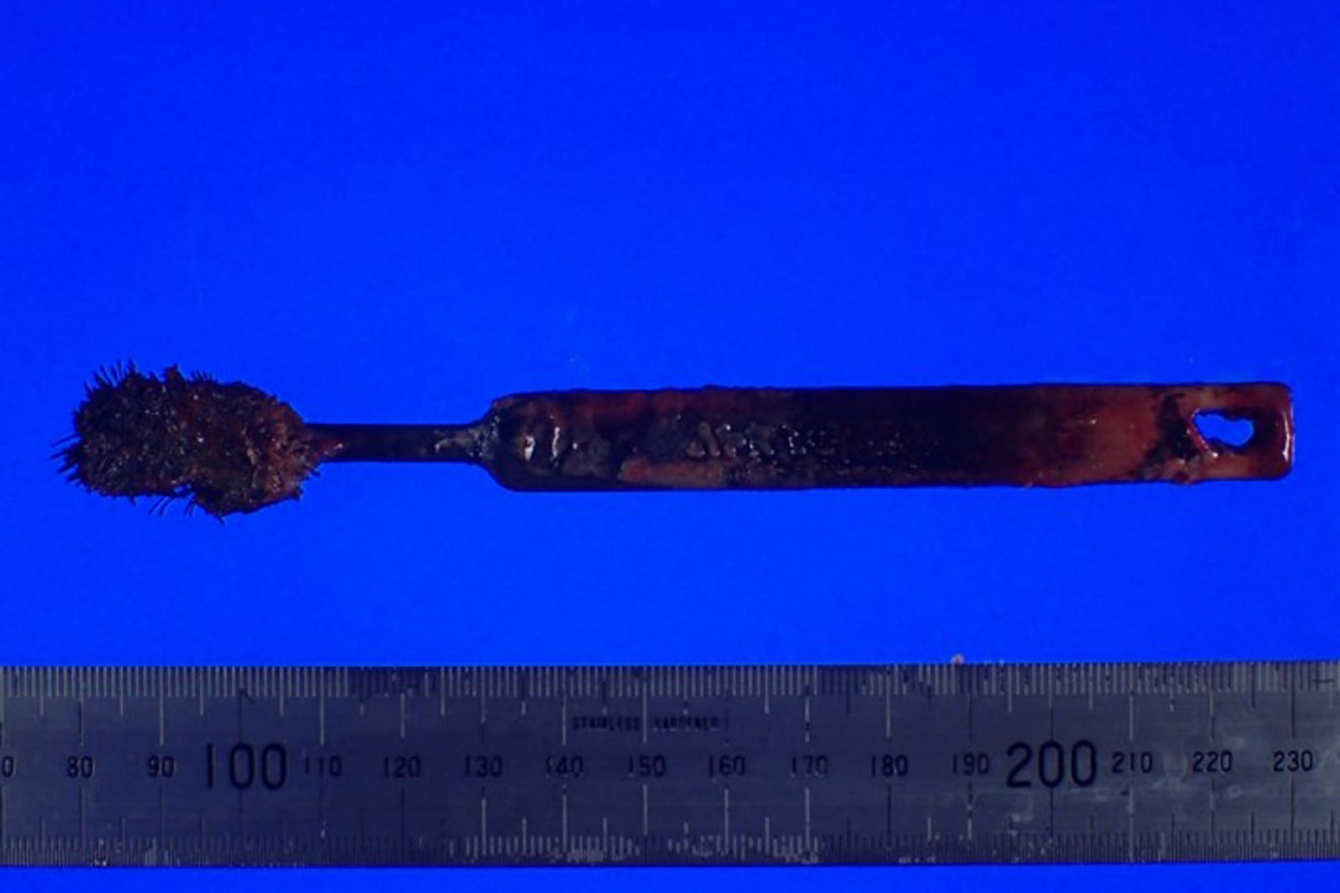
The extracted toothbrush was 15 cm in length

A 51-year-old male with intellectual disability presented to our hospital due to fever. He denied any pain or other symptoms, and his vital signs were normal. Laboratory values were as follows: 11,500/mm^3^ white blood cells, 12.3 g/dL hemoglobin, 5 mg/L C-reactive protein, 12 U/L aspartate aminotransferase, and 9 U/L alanine aminotransferase. Initially, he was diagnosed with aspiration pneumonia based on chest X-ray and blood examination. However, an abdominal X-ray examination suggested a foreign body (Fig. 1a), and a computed tomography (CT) scan revealed a toothbrush in the duodenum (Fig. 1b). No abnormal ascites fluid, free air, and abdominal abscess were observed, and it looked as if the toothbrush was stuck in the liver. An upper gastrointestinal endoscopy was performed immediately following the CT studies. It was observed in the second part of duodenum, and there was a granuloma around the handle part of the toothbrush at the duodenal bulb (Fig. 2). Endoscopic removal was attempted using a polypectomy snare and biopsy forceps. However, the toothbrush was deeply embedded into the duodenal mucosa, so it could not be safely removed. This case was diagnosed as duodenal incarceration of the toothbrush, and it was removed by laparoscopic surgery, which is less invasive than open surgery. The surgical procedure was as follows (Fig. 3a). First, we found that the hepatic hilum and duodenal bulb were adhered tightly and could not be detached by peeling. Therefore, the toothbrush was difficult to remove by making an incision in the duodenal bulb. For that, the transverse and ascending colons were detached from the retroperitoneum to expose the C-loop of the duodenum in which the toothbrush was incarcerated. After that, an incision was made on the caudal side of the second part of the duodenum, and the toothbrush was removed through the incision (Fig. 3b). The extraction hole was closed using a barbed suture, and the procedure was completed. The operation time and estimated blood loss were 201 min and a little, respectively. The extracted toothbrush was 15 cm in length (Fig. 4). The patient’s postoperative course was uneventful without complications and a postoperative CT showed no changes in the liver (Fig. 1c). During the postoperative course, no abnormal values of liver function were observed; these results were consistent with those of the preoperative blood examination.

## Discussion

Most foreign body intakes are accidental, but several factors are associated with this. The majority of foreign body ingestions occur in children because of carelessness. In adults, foreign body intake is commonly encountered in patients with intellectual disabilities, psychosis, or alcoholism, and in the elderly wearing dental prostheses [3]. The most common causes are mental disorders (bulimia nervosa/anorexia nervosa, schizophrenia) and suicide [4]. In our case, the patient had a past history of congenital developmental disorder, which may have been the cause of toothbrush ingestion. Until now, the accidental ingestion of various foreign bodies, such as toothpicks, fish and meat bones, screws, coins, metal clips, teeth, dentures, and spoon handles, has been reported [3, 5, 6]. Even if small foreign bodies like these were swallowed, swallowing an entire toothbrush is a rare event [7], and less than 50 cases were reported to have swallowed a toothbrush in 2022 [2, 8].

Most ingested foreign bodies pass through the gastrointestinal tract spontaneously (80–90%) [3, 6, 9], but large or long foreign bodies, such as toothbrush, can be difficult to pass through the entire gastrointestinal tract. This is because after the lower esophageal sphincter, there are three physiological narrowing in the gastrointestinal tract, which are the pylorus, duodenal C-loop, and ileocecal junction. There were also cases in which a toothbrush advanced to the duodenum and ascending colon in the gastrointestinal tract [2, 10]. However, as of today, there is no documentation described that a toothbrush passed through the entire gastrointestinal tract and was naturally eliminated [8]. The main complications caused by foreign body ingestion are obstruction, pressure necrosis, mucosal tear, hemorrhage, and gastrointestinal tract perforation [2]. Although these complications may occur in all segments of the gastrointestinal tract [4], the ileum is considered the most common perforation site [11]. To date, perforations in the duodenum [12], ileum [13], and ascending colon [14] have been reported.

Therefore, to avoid these complications, we should get rid of ingested foreign bodies if they cannot pass through the digestive tract naturally. Furthermore, a prompt intervention is required to avoid these critical complications, including a massive hemorrhage and gastrointestinal tract perforation. The European Society for Gastrointestinal Endoscopy recommends an emergency endoscopy for foreign bodies in the stomach, such as sharp objects, magnets, batteries, and large and long objects, within 24 h. For dull foreign bodies in the stomach, a non-urgent therapeutic endoscopy within 72 h is recommended [15]. The first successful performance of endoscopic removal of toothbrush was reported by Ertan et al. [16], along with other reports [17, 18]. If a foreign body cannot be removed by endoscopy, another approach is required depending on its size and type. Approximately 10–20% of cases of foreign body ingestion require endoscopic removal, while less than 1% need surgery for foreign body extraction or to treat its complications [18]. There are also some reports wherein a toothbrush in the stomach was removed by laparotomy [19]. Wishner also reported a successful case of laparoscopic removal of a toothbrush from the stomach [20]. This approach was recommended by the author to remove ingested foreign bodies because it is minimally invasive [20]. However, as far as we know, there are no reports of laparoscopic removal of duodenal foreign bodies.

Herein, we tried to remove the toothbrush in the duodenum by endoscopy, but it could not be removed due to its length and hardness. Therefore, when a surgical intervention was required for its removal, laparoscopic surgery was selected because it is minimally invasive. Our surgery teams, who are proficient in laparoscopic surgery, were able to perform it safely and reasonably using their anatomical knowledge and laparoscopic surgery techniques necessary for gastric cancer surgery and colorectal cancer surgery.

## Conclusion

We experienced a rare case of a duodenal foreign body, which was a toothbrush. Surgical intervention was required because it was difficult to remove by endoscopy, so laparoscopic surgery was performed. To the best of our knowledge, this is the first report of laparoscopic removal of a toothbrush in the duodenum. We believe that this laparoscopic approach may be an alternative to traditional laparotomy for duodenal foreign bodies if an endoscopic removal is not possible.

## Data Availability

The dataset supporting the conclusions of this article is available in the manuscript.

## Abbreviation

CT: Computed tomography
